# Role of the Peripheral Nervous System in PD Pathology, Diagnosis, and Treatment

**DOI:** 10.3389/fnins.2021.598457

**Published:** 2021-04-30

**Authors:** Chengxiao Ma, Wen Zhang, Maohong Cao

**Affiliations:** ^1^Department of Neurology, Affiliated Hospital of Nantong University, Nantong, China; ^2^Research Center of Clinical Medicine, Affiliated Hospital of Nantong University, Nantong, China; ^3^Department of Neurology, Affiliated Hospital of Nantong University, Nantong, China

**Keywords:** Parkinson’s disease, dopaminergic neuron, peripheral nervous, Schwann cell, graft

## Abstract

Studies on Parkinson disease (PD) have mostly focused on the central nervous system—specifically, on the loss of mesencephalic dopaminergic neurons and associated motor dysfunction. However, the peripheral nervous system (PNS) is gaining prominence in PD research, with increasing clinical attention being paid to non-motor symptoms. Researchers found abnormal deposition of α-synuclein and neuroinflammation in the PNS. Attempts have been made to use these pathological changes during the clinical diagnosis of PD. Animal studies demonstrated that combined transplantation of autologous peripheral nerves and cells with tyrosine hydroxylase activity can reduce dopaminergic neuronal damage, and similar effects were observed in some clinical trials. In this review, we will systematically explain PNS performance in PD pathology and its clinical diagnostic research, describe PNS experimental results [especially Schwann cell (SC) transplantation in the treatment of PD animal models] and the results of clinical trials, and discuss future directions. The mechanism by which SCs produce such a therapeutic effect and the safety of transplantation therapy are briefly described.

## Introduction

Parkinson disease (PD) is a typical neurodegenerative disorder of the nervous system. A pathologic hallmark of PD is the presence of intracytoplasmic inclusions known as Lewy bodies (LBs) composed of α-synuclein aggregates in neurons in the pars compacta of the substantia nigra (SN). The 6-stage theory proposed by Braak to explain the pathology and clinical development of PD postulates that neurodegeneration begins in the peripheral nervous system (PNS), with progressive involvement of the central nervous system (CNS) from caudal to rostral brain areas and corresponding clinical manifestations (Braak et al., 2003; Braak and Del Tredici, 2017). This hypothesis has been substantiated by studies demonstrating α-synuclein deposition in the gastrointestinal nervous system (Qualman et al., 1984; Kupsky et al., 1987; Wakabayashi et al., 1988; Iranzo et al., 2014) and cardiac denervation detected by imaging preceding the loss of dopaminergic neurons in the SN (Salsone et al., 2012). Although it cannot fully explain the clinical course of PD, this theory provides a framework for studying of PD and has yielded important insights into the disease.

**FIGURE 1:**
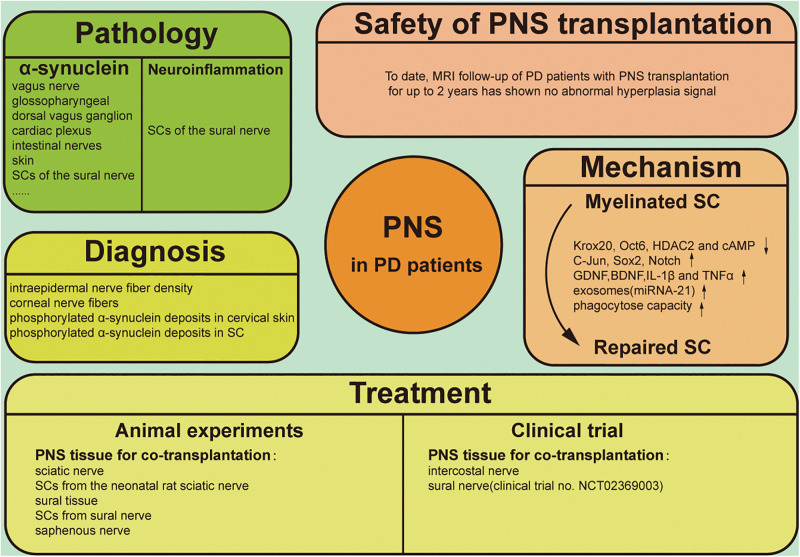
Graphical illustration of PNS involving the pathology, diagnosis, treatment, therapeutic mechanism and safety of clinical application in PD.

The clinical diagnosis of PD is mostly based on motor symptoms, which may not appear until there is damage to 30–50% of dopaminergic neurons in the brain (Fearnley and Lees, 1991; Ma et al., 1997; Lang and Lozano, 1998). As such, early diagnosis of PD is critical for preserving the integrity of motor neurons and related motor functions. Although there are no reliable blood biomarkers for PD, imaging of dopaminergic neurons is a reliable method for detecting early-stage PD. Positron emission tomography (PET) and single-photon emission computed tomography (SPECT) can reveal the density of presynaptic terminals of SN dopaminergic neurons projecting into the striatum for early and accurate assessment of disease progression (Brooks and Pavese, 2011). Meanwhile, new treatment methods such as transplantation of induced pluripotent stem cells are being investigated (Kikuchi et al., 2017; Kikuchi et al., 2018), although their safety and efficacy require further assessment, and ethical concerns must be addressed (Yasuhara et al., 2017; Sonntag et al., 2018). The diagnosis and treatment of PD based on CNS manifestations does not take into account all aspects of the disease, and increasing attention has been paid to PNS involvement (Wakabayashi et al., 2010; Comi et al., 2014). Specifically, Schwann cells (SCs) have been studied for their potential to support injured neurons and promote neuroregeneration (Kim et al., 2013; Brosius Lutz and Barres, 2014; Jessen et al., 2015). SCs have also been linked to pathologic changes in PD and may be important for its clinical diagnosis as well as treatment (Tu et al., 1998; Timmer et al., 2004; Zhang et al., 2019). This review discusses PNS manifestations in PD, with a focus on the role of SCs in the pathologic changes associated with the disease and their potential application to PD diagnosis and treatment (Figure 1).

## Pathologic Changes in the PNS in PD

Parkinson disease is a multisystem disease with numerous clinical manifestations. In addition to the typical motor symptoms, there are many non-motor symptoms such as skin sensory and olfactory abnormalities and autonomic dysfunction, which are accompanied by aberrant protein deposition in the PNS. In PD patients with dysphagia, α-synuclein aggregates have been detected in the pharyngeal motor and sensory branches of the vagus nerve, as well as in the glossopharyngeal and internal superior laryngeal nerves (Mu et al., 2013a,b). LBs have also been found in the dorsal vagus ganglion (Jackson et al., 1995; Kövari et al., 2007), parasympathetic sacral nuclei (Bloch et al., 2006), cardiac plexus (Orimo et al., 2008; Salsone et al., 2012), and esophageal and intestinal nerves (Qualman et al., 1984; Kupsky et al., 1987; Wakabayashi et al., 1988; Iranzo et al., 2014). Bilateral vagus nerve transection was shown to block the spread of α-synuclein from the gut to the brain and prevent neurodegeneration (Kim et al., 2019), supporting Braak’s theory that PD spreads from the PNS to the CNS. LBs have also been found in the skin of PD patients (Dabby et al., 2006; Ikemura et al., 2008; Miki et al., 2010; Doppler et al., 2014; Sharma, 2014); in 279 postmortem specimens of the abdominal wall and upper arm skin, immunopositivity for phosphorylated α-synuclein was detected in unmyelinated fibers of the dermis in 20 of 85 patients with LB pathology in the CNS and adrenal glands, while the remaining 194 patients without LB pathology had negative immunoreactivity. A retrospective analysis of 142 patients with PD found that the sensitivity of this skin test was ∼70% (Ikemura et al., 2008); however, in a clinical trial, the detection rate of cutaneous α-synuclein was just 10%. In 2 out of 20 patients with confirmed PD, a skin test revealed abnormal α-synuclein accumulation in non-medullated fibers of the anterior chest skin. The loss of peripheral nerve fibers is an inherent feature of PD that reflects two aspects of abnormal α-synuclein aggregation in the CNS: axonal degeneration and neuronal death. Thus, detection of phosphorylated α-synuclein in dermal nerve fibers may be a highly specific method for PD diagnosis, albeit with low sensitivity (Doppler et al., 2014).

Schwann cells are abundant in the PNS, and their density around myelinated axons is markedly reduced in PD patients (Kanda et al., 1996). α-Synuclein immunoreactivity was observed in SCs near spinal nerve roots but not in the myelin sheath or axons; moreover, astrocytes and oligodendrocytes were positive for α-synuclein expression but oligodendrocyte progenitor cells were negative (Papadopoulos et al., 2006). A recent clinical study reported that phosphorylated α-synuclein deposits were present in SCs of the sural nerve in PD patients but almost absent in axons (Zhang et al., 2019). SCs with cytoplasmic structures containing phosphorylated α-synuclein have also been observed in patients with multiple system atrophy (MSA) (Nakamura et al., 2015). Thus, SCs are implicated in neurodegenerative diseases characterized by abnormal α-synuclein accumulation and may serve as a pathologic marker for disease diagnosis.

Neuroinflammation is an important pathological manifestation of PD, and reactive microglia were first found in the brains of PD autopsy cases over 30 years ago (McGeer et al., 1988). Microglia can scavenge abnormal α-synuclein produced by neurons and produce a variety of pro-inflammatory factors, such as interleukin (IL)-1β, IL-6, and tumor necrosis factor-α (TNF-α) in the brain and cerebrospinal fluid (CSF) of patients (Harms et al., 2021). The researchers also found large numbers of activated microglia in areas outside the SN, and this activation was not directly linked to the presence of LBs, suggesting that the role of microglia in PD neuroinflammation goes beyond the scavenging of abnormal proteins and dead neurons (Imamura et al., 2003). Axonal degeneration of peripheral nerves and activation of SCs with secretion of multiple inflammatory factors are also observed in PD patients, but these lesions are not necessarily associated with sensory abnormalities in patients (Zhang et al., 2020). Welleford et al. performed RNA sequencing on the sural nerve of six patients undergoing DBS surgery. They first intercepted a section of the sural nerve. Two weeks after the initial injury, another section of the nerve fascicles of the distal and pre-degenerated stump of the nerve was dissected and processed for RNA-sequencing studies. The results showed that SCs upregulated the expression of genes related to dedifferentiation, immunity, and growth, such as IL-6, IL-10, leukemia inhibitory factor (LIF), and glial cell-derived neurotrophic factor (GDNF) significantly increased (Welleford et al., 2020). Collectively, the evidence indicates that SCs are involved in PD neuroinflammation.

## PNS in the Diagnosis of PD

Peripheral nervous system manifestations of PD are useful for adjunctive diagnostic screening, and skin biopsies are widely used for diagnosis (Table 1). Intraepidermal nerve fiber density was lower in PD patients than in healthy subjects, with a greater reduction on the more severely affected side that was accompanied by localized skin sensory and tactile abnormalities (Lin et al., 2016; Nolano et al., 2017; Jeziorska et al., 2019). Another study confirmed that the densities of intraepidermal and corneal nerve fibers were significantly reduced in PD patients compared to normal subjects (Kass-Iliyya et al., 2015). Notably, epidermal nerve fiber density and autonomic innervation were significantly reduced in idiopathic (I) PD patients, while skin biopsies in patients with parkinsonism were normal (Giannoccaro et al., 2015). IPD patients also had small nerve fiber lesions in the skin of the legs and phosphorylated α-synuclein deposits in cervical skin, whereas patients with parkinsonism of different etiologies were presumed to lack synuclein deposits and had normal nerve fibers, with no pathologic protein deposits (Donadio et al., 2014). Phosphorylated α-synuclein was mostly deposited in SCs and largely absent in the axons (Zhang et al., 2019). It should be noted that α-synuclein deposition is not an exclusive feature of PD; it is also observed in patients with dementia with LBs and MSA (Tu et al., 1998; Giasson et al., 2000; Wakabayashi et al., 2002; Nishie et al., 2004; Foulds et al., 2012) and therefore cannot be used on its own to diagnose PD.

**TABLE 1:** Application of PNS in clinical diagnosis and trials.

**Authors**	**Patient**	**Diagnosis method**	**Outcomes**
**Clinical diagnosis**			
Lin et al., 2016	Twenty-eight PD patients	Skin biopsy, contact heat-evoked potential (CHEP)	PD patients had reduced intraepidermal nerve fiber density and CHEP amplitude
Jeziorska et al., 2019	Twenty-three PD patients	Skin biopsy	Intraepidermal nerve fiber density and subepidermal nerve fiber length were lower in more affected versus less affected side
Nolano et al., 2017	Fifty-four PD patients	Skin biopsy	Intraepidermal nerve fiber density was lower in patients and the loss of it was higher in the more affected side
Kass-Iliyya et al., 2015	Twenty-six PD patients	Corneal confocal microscopy (26/26), skin biopsy (24/26)	PD patients had significantly reduced in corneal nerve fiber density and intraepidermal nerve fiber density
Giannoccaro et al., 2015	Twenty-two idiopathic Parkinson disease (IPD), and eleven parkinsonism patients	^123^I-MIBG myocardial scintigraphy and Skin biopsy	In the IPD group, both ^123^I-MIBG scintigraphy and skin biopsy results were abnormal in 91% of patients. In parkinsonism, results of both tests were normal in all patients
Donadio et al., 2014	Twenty-one IPD and twenty parkinsonism patients	Skin biopsy	IPD patients showed a small nerve fiber neuropathy prevalent in the leg with phosphorylated a-synuclein deposited in the cervical skin. Parkinsonism patients did not show these signs
Zhang et al., 2019	Sixteen PD patients	Sural nerve biopsy	Deposition of phosphorylated α-synuclein was found in 16/16 PD patients

**Authors**	**Patient**	**Implantation method**	**Outcomes**

**Clinical trials**			
Date et al., 1995	A 55-year-old woman and a 43-year-old patient with advanced Parkinson’s disease	Co-grafts of adrenal medulla and peripheral nerve into the bilateral caudate nuclei	Both patients showed improvement in PD symptoms after transplantation
Watts et al., 1997	Five patients with advanced Parkinson’s disease	Patients received unilateral intrastriatal adrenal medulla-intercostal nerve co-grafts	The clinical improvement from this procedure is sustained for 24 months
López-Lozano et al., 1999	Four patients PD	The adrenal medulla and intercostal nerve were implanted into right caudate nucleus	Improved symptoms in on and off phases persist in all four cases
Nakao et al., 2004	Four patients with PD	Thoracic sympathetic ganglia into the brain	Two fold increase in the duration of the “on” phase induced by a single dose of levodopa
van Horne et al., 2018	Eight patients with a diagnosis of idiopathic PD	Patients receive bilateral DBS and unilateral segments of the sural nerve of the STN	The lateralized UPDRS scores showed a more significant overall reduction in scores on the side contralateral to the graft

## Applications of PNS in PD Treatment

### Evidence for SC Involvement in PD From Animal Models

Although the relationship between pathologic changes and PD progression is not fully understood, the therapeutic potential of SCs for PD has been widely investigated in animal experiments based on their neuroregenerative capacity in the PNS (Table 2). In the 1980s, Aguayo and colleagues demonstrated that PNS grafts could support CNS neuron survival following injury and guide axonal regeneration in mice. Transplanted dopaminergic neurons survived and extended fibers into a peripheral nerve bridge formed by homotopic sciatic nerve covering the skull that connected the midbrain tissue graft over the superior colliculus and the caudate-putamen nucleus (Aguayo et al., 1984).

**TABLE 2:** Application of PNS in animal experiments.

**Authors**	**Animal model**	**Graft**	**Outcomes**
**Animal test**			
Aguayo et al., 1984	6-OHDA lesioned right nigrostriatal of Female Sprague-Dawley rats	Fetal mesencephalic and heterologous sciatic nerve	Monoaminergic neurons within the implant extended axons along the entire length of the nerve bridges and some of these fibers extended into the striatum
Date et al., 1990	MPTP-treated male C57BL/6 mice	Adrenal medullae and sciatic nerve	The co-grafted mice showed a better survival of adrenal medullary chromaffin cells and longer fibers of host DA neurons
van Horne et al., 1991	6-OHDA lesioned male Fisher-344 rats	Fetal ventral mesencephalic and sciatic nerve	The co-graft group revealed a significantly more significant decrease in rotation than the VM group
Wilby et al., 1999	6-OHDA lesioned female rats	SCTM41 ± GDNF and nigra graft	Co-grafts improved the survival of intrastriatal embryonic dopaminergic neuronal grafts. Bridge grafts promoted the growth of axons through the grafts to the striatum
Timmer et al., 2004	6-OHDA lesioned medial forebrain bundle (MFB) of female Sprague–Dawley rats	Ventral mesencephalic tissue and Schwann cells	21/23 kDa FGF-2-secreting SCs promoted the survival of dopaminergic neurons
Kordower et al., 1990	MPTP lesioned aged female rhesus monkeys	Autologous adrenal chromaffin cells and sural nerve	Co-grafted chromaffin cells exhibited multipolar neuritic processes and numerous chromaffin granules
Watts et al., 1995	Right hemi parkinsonian by left intracarotid injection of MPTP	Autologous adrenal chromaffin cells and sural nerve	Animals undergoing autologous co-grafts demonstrated improved motor performance than the control animal
Xia et al., 2012	6-OHDA lesioned right caput nuclei caudate and right dorsal caudate putamen	SCs and NSCs	Co-transplantation of SCs and NSCs could effectively cure PD in macaques
Collier et al., 1994	MPTP lesioned adult male St Kitts African Green monkeys	Monkey saphenous nerve and embryonic ventral mesencephalic tissue	Morphological observations indicated that no evident augmentation of the morphology of grafted dopamine neurons
Howel et al., 2000	MPTP-induced hemi parkinsonian model in rhesus monkeys	Adrenal chromaffin cells and sural nerve	Recovery of behavioral function after surgical treatment, with adrenal co-grafted monkeys showing the highest degree of improvement

To investigate the influence of the CNS environment on peripheral neuron grafts, one study compared the therapeutic efficacy of adrenal medullary cells transplanted alone or with sciatic nerve fragments into the striatum of mice with 1-methyl-4-phenyl-1,2,3,6-tetrahydropyridine-induced PD (Date et al., 1990). The results showed that the number of tyrosine hydroxylase (TH)-positive chromaffin cells, density of TH fibers, and concentration of dopamine in the brain were higher in the sciatic nerve group. Similarly, grafting of the sciatic nerve along with embryonic midbrain tissue in a rat model of 6-hydroxydopamine (6-OHDA)-induced PD resulted in greater improvement in an apomorphine-induced rotation test compared to the monograft group (van Horne et al., 1991). Immunofluorescence analysis revealed the secretion of basement membrane components by SCs, but an immune response was rarely observed. Collectively, these results suggest that PNS grafts can survive in the CNS and can promote the orderly growth of new nerve fibers, with consequent functional improvements.

In animal experiments where PNS fragments were transplanted into the brain for therapeutic purposes, SCs were identified as the functional component. Direct grafting of SCs is another possible approach for neuronal regeneration in the brain. To this end, GDNF-secreting SCs were engineered by lentiviral transduction from neonatal rat sciatic nerve cultures purified from SCs (SCTM41) (Wilby et al., 1999). Transplantation of either SCTM41 or SCTM41-GDNF improved the survival of intrastriatal embryonic dopaminergic neuron grafts and promoted neurite outgrowth into the host neuropil, although SCTM41-GDNF had a more potent effect. In a bridging experiment, both types of SCs induced axonal growth of grafted cells into the striatum, with SCTM41-GDNF being more effective both in terms of the density and total number of TH-positive axons. In another study, modification of SCs to secrete basic fibroblast growth factor-2—a cytokine with mitogenic and pro-survival activities—promoted graft survival and neuronal growth in the CNS (Timmer et al., 2004).

Primate studies are necessary to verify the clinical applicability of SCs to treat PD. When equal amounts of autologous adrenal medulla and sural tissue were transplanted into the brains of rhesus monkeys, the graft survival rate was 4–8 times higher than with adrenal medulla transplantation alone (Kordower et al., 1990). Moreover, medullary cells in the co-transplant group had more chromaffin granules and neurites that formed synaptic connections with surrounding axons. These findings were substantiated by a similar study (Watts et al., 1995). Rhesus monkeys with 6-OHDA-induced injury transplanted with neural stem cells from aborted fetal mesencephalic tissue in combination with SCs showed improved motor function 1 month later, with recovery of fine motor control after 4 months; PET scanning revealed accumulation of ^18^F-FP-β-CIT (a radioligand used for dopamine transporter quantification) in the injured striatum (Xia et al., 2012). However, cell-based therapy in primates has not been as effective as anticipated. For instance, there was no significant functional improvement when a hollow tube with a semi-permeable polymer as a carrier was used to insert a saphenous nerve segment into the lateral ventricle, with transplantation of monkey embryo-derived mesencephalon tissue into the caudate nucleus 2 mm rostral and 2 mm caudal to the polymer implant (Collier et al., 1994). Although the authors claimed that this experimental strategy avoided the brain tissue damage associated with conventional transplantation, spatial separation between the transplanted and native tissues may have prevented successful graft integration. Monkeys treated by co-transplantation of sural nerve and an adrenal graft showed greater behavioral improvements than the surgical control; however, the 3,4-dihydroxyphenylacetic acid and homovanillic acid levels in CSF were not different between the two groups after 10–12 months (Howel et al., 2000). Moreover, there was significant interindividual variability in fine hand movement recovery, which precluded group analysis. This problem is rarely encountered in behavioral studies using rodents and highlights the complexity of primate behavior. Given the importance of primate experiments for developing new therapies, more rational designs for behavioral experiments and refinement of pathophysiologic tests are needed.

### Personalized PD Therapy Using SCs

Schwann cells have been used to treat spinal cord injury (Saberi et al., 2008, 2011; Yazdani et al., 2013; Oraee-Yazdani et al., 2016; Anderson et al., 2017), but few trials have investigated their use in PD (Table 1). Several trials conducted in the 1990s showed that adrenal medulla transplantation combined with peripheral nerve grafting alleviated motor impairment (Date et al., 1995, 1996; Watts et al., 1997; López-Lozano et al., 1999). These studies used autologous sympathetic nerves as grafts and reported a significantly longer “on” period in four patients during follow-up. At the 1-year follow-up of eight PD patients who underwent deep brain stimulation (DBS) of the subthalamic nucleus with concurrent autologous sural nerve grafting in the SN, the Unified Parkinson’s Disease Rating Scale III (UPDRs-III) scores decreased from 32.5 ± 9.7 at baseline to 25.1 ± 15.9, with a more prominent reduction for the contralateral limb. The complications associated with obtaining the autologous sural nerve were that three participants described a patch of numbness on the lateral aspect of the foot. This phenomenon was also manifested in some patients who took biopsy of the sural nerve, and one patient developed local superficial cellulitis and recovered after antibiotic treatment. Postoperative magnetic resonance imaging (MRI) showed there was no significant edema or hemorrhage in the graft target zone (van Horne et al., 2018). The number of patients in these trials was low, but a phase 1 clinical trial has been initiated with an estimated enrollment of 72 patients (clinical trial no. NCT02369003). The study started in February 2015 and plans to complete the primary outcome in September 2020. Adverse events will be collected to measure the safety and tolerability of the grafting procedure. Dopamine neurodegeneration at 12 or 24 months will be assessed using SPECT imaging and compared to scans obtained taken before DBS surgery. This trial will investigate whether autologous SCs are effective in repairing lesioned dopaminergic neurons in the CNS. If successful, it would herald the development of a new treatment for PD in which patients can provide their own tissue as a source of growth factors that could arrest or reverse the ongoing cellular loss underlying their devastating dysfunction.

There are some outstanding issues that must be addressed regarding the therapeutic application of autologous SCs. First, the intercellular interactions and molecular mechanisms of SCs in promoting neuronal regeneration remain to be elucidated. Secondly, LBs have been detected in the SCs of PD patients, calling into question the safety of using autologous SCs for cell-based therapy. On the other hand, clinical trials have shown that such pathologic alterations do not influence the therapeutic effects of SCs in CNS; in fact, α-synuclein accumulation may activate SCs, thereby enhancing nerve repair.

## SC Mechanisms for Neuronal Repair and Regeneration

Schwann cells are a type of peripheral glial cell originating from the neural crest that initially differentiate into SC precursor cells, then immature SCs, and finally into myelinating and non-myelinating cells that retain the ability to dedifferentiate to an immature SC state (Bhatheja and Field, 2006; Griffin and Thompson, 2008; Jessen et al., 2015). SCs secrete a basal lamina composed of growth-promoting laminin, type IV collagen, and heparin sulfate proteoglycans (Bunge et al., 1990), which are critical for the SC myelinating function. More importantly, SCs proliferating after nerve injury may form a channel that promotes axonal regeneration along residual SC structures known as Büngner bands (Chen et al., 2007; Faroni et al., 2015). In contrast, oligodendrocytes in the CNS do not secrete basal lamina, so the healthy CNS is free of these basal lamina components except at the pial surface and sites of contact between astrocytes and blood vessels (Squire et al., 2009; Martini et al., 2010). SC maturation is accompanied by the establishment of autocrine circuits involving platelet-derived growth factor, insulin-like growth factor-1, and neurotrophin-3 that allow SCs to survive after nerve injury and promote peripheral nerve regeneration (Jessen and Mirsky, 1999). Up to 30 or 40% of oligodendrocytes may be lost following CNS injury, and those that survive may be unable to support neuroregeneration (Ludwin, 1990). SC autocrine and cytokine secretion functions are not limited to the PNS, as demonstrated in animal models and clinical trials of SC transplantation into the brain.

Exosomes are secretory vesicles containing mRNA and microRNA secreted by cells, and they have a variety of biological effects. It is thought that supplementing their natural function could enable targeted delivery of drug molecules (Mehryab et al., 2020). Multiple studies have confirmed that SC can secrete exosomes and that they have a powerful promotion effect on neuronal axon regeneration and improve neuronal viability (Hu et al., 2019; Yu et al., 2021). It was found that miRNA-21 expression was increased in secreted exosomes after SC upregulated the expression of c-jun and Sox2, which are key in promoting neurite growth by SC-derived exosomes (López-Leal et al., 2020). Glutamate and calcium ions may also play a role in this process (Hu et al., 2019).

**FIGURE 2:**
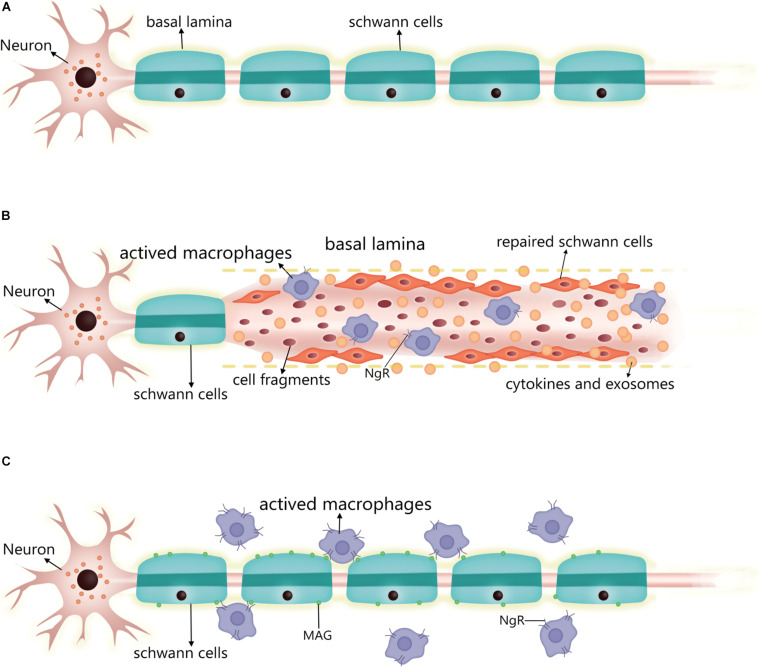
Changes in SC after PNS injury and the performance of the nerve repair process. **(A)** Normal myelinated SC and basal lamina formation. **(B)** Post-injury, residual SCs upregulate repair-related signals into repaired SCs, which can align along the residual basal lamina to guide neuronal axon growth, while secreting cytokines and exosomes to promote neuronal survival and neurite growth. The repaired SCs also enhance phagocytosis to scavenge cell fragments, secreting inflammatory factors to regulate surrounding inflammation and recruiting macrophages into the injury to enhance cell fragment clearance. At this stage, the macrophage surface expresses a small amount of NgR. **(C)** After the repair is complete, the SC re-formed myelin sheath wraps around the axon and produces a basal lamina. The SC expresses MAG, which interacts with the macrophage surface NgR to induce the macrophage to migrate out of the nerve. At this stage, macrophage expression of NgR is significantly increased. SC, Schwann cell; PNS, peripheral nervous system; NgR, Nogo receptor; MAG, myelin-associated glycoprotein.

Following neuron injury (Figure 2), SCs phagocytose cell fragments and secrete inflammatory factors such as TNF-α, LIF, IL-1, and IL-6 that recruit other phagocytes (Kass-Iliyya et al., 2015; Kikuchi et al., 2018). Fatty acids released during myelin breakdown also regulate the inflammatory response by producing prostaglandins and leukotrienes that facilitate immune cell penetration into damaged nerve tissue (Martini et al., 2008). Phagocytosed SC-derived myelin fragments induce macrophage differentiation toward an anti-inflammatory phenotype (Boven et al., 2006). SCs induce the egress of macrophages out of Büngner bands during subsequent myelination by interacting with the macrophage Nogo receptor (NgR) and myelin-associated glycoprotein (Fry et al., 2007; David et al., 2008). If these macrophages are not cleared, their prolonged residence in the nerve can lead to chronic inflammation and nerve damage. Thus, following axonal injury, surviving SCs secrete cytokines and chemokines that prevent further neuronal damage, participate in postinjury debris removal, recruit macrophages that participate in debris clearance, and modulate the local inflammatory response.

The myelinated SC is transformed into a repair state after injury in two steps. The expression of myelination-related signaling molecules such as Krox20, Oct6, HDAC2, and cAMP is downregulated, while that of repair-related signaling molecules such as c-jun, Sox2, and Notch is promoted (Jessen and Arthur-Farraj, 2019). c-Jun is a key regulator in the SC injury response. After injury, it is rapidly upregulated and negatively regulates the myelin program, and promotes expression of the repair program (Arthur-Farraj et al., 2012; Fazal et al., 2017). Additionally, zinc finger E-box binding homeobox (Zeb) 2 has been identified as an essential regulator of SC differentiation, myelination, and nerve repair (Quintes et al., 2016). The timing of the expression of these reparative molecules differed after injury, with cytokine expression (e.g., IL-1β and TNFα) showing upregulation 1 day after injury, GDNF expression peaking at about 1 week, brain-derived neurotrophic factor expression peaking at 2–3 weeks, and c-jun also being rapidly expressed after injury and lasting at least 7–10 days (Rotshenker, 2011; Jessen and Mirsky, 2019). Moreover, SCs were also found to upregulate some inflammation-related signaling molecules and secrete pro-inflammatory factors to regulate the inflammatory response at the affected site 2 weeks after the injury (Kikuchi et al., 2018; Welleford et al., 2020). In conclusion, the transition process of SCs to the repair form after injury is very complex and regulated by multiple factors.

## Safety of Cell-Based Therapy

There are strict ethical and safety requirements for human cell transplantation therapy. Animal-derived mesencephalic tissue is commonly used in animal experiments but has little possibility of being used in clinical settings. Even the use of human embryonic stem cells from aborted fetuses for clinical purposes is hampered by tremendous ethical obstacles. Therefore, donor tissue is usually derived from patients themselves, which has the advantage of a reduced risk of graft rejection.

A major concern in cell-based therapy is the possibility that autologous cells can grow and differentiate into neoplasms in a non-native environment. In one case study of a 43-year-old female patient, cranial MRI at 1, 12, and 24 months following autologous peripheral nerve transplantation showed signal enhancement at the graft site but there were no abnormal signals in other brain regions (Date et al., 1995). In a similar study, no abnormal hyperplasia, cerebral infarction, or cerebral hemorrhage was detected on MRI of the graft site 1 year after transplantation surgery (van Horne et al., 2018). Although these results are encouraging, they do not provide sufficient evidence for the safety of peripheral neuron grafting in the treatment of PD, given the paucity of cases. However, relevant insight can be garnered from studies in which peripheral neurons were used for the treatment of injured spinal cord, which is part of the CNS. In eight patients with chronic spinal cord injury who were transplanted with mesenchymal stem cells combined with SCs, MRI examination at 6, 12, and 18 months postsurgery showed no tumor-like tissue growth (Yazdani et al., 2013). Similarly, in six patients with subacute spinal cord injury treated with autologous SCs of sural nerve origin, there was no MRI evidence of hyperplasia up to 12 months later (Anderson et al., 2017).

## Conclusion

The PNS is relevant to the diagnosis and treatment of PD, but its clinical application has not been fully exploited. For instance, it may be possible to develop a compound that can transiently chelate α-synuclein *in vivo* with no harm or low toxicity to humans, and detection of this complex by radiographic or other imaging methods could provide a diagnostic tool for PD and other neurodegenerative diseases characterized by abnormal protein aggregation. PD treatment should also be differentiated according to the disease stage, in accordance with Braak’s six-stage theory. There are no incompatibilities between drugs, DBS, and cell-based therapy in the treatment of PD, but the timing of each intervention may be critical for maximizing efficacy. Intracerebral therapy by transplantation of autologous SCs derived from the PNS is a promising therapeutic strategy that can potentially prevent or slow PD progression. For this approach to be successful, basic questions such as the mechanisms underlying the interaction between a patient’s own SCs and brain dopaminergic neurons must be answered.

## Author Contributions

MC designed the study. CM and WZ reviewed the literature. CM wrote the manuscript. All authors contributed to the article and approved the submitted version.

## Conflict of Interest

The authors declare that the research was conducted in the absence of any commercial or financial relationships that could be construed as a potential conflict of interest.
